# Introducing the ESAT-6 free IGRA, a companion diagnostic for TB vaccines based on ESAT-6

**DOI:** 10.1038/srep45969

**Published:** 2017-04-07

**Authors:** Morten Ruhwald, Lena de Thurah, Davis Kuchaka, Mostafa Rafaat Zaher, Ahmed M. Salman, Abdel-Rahman Abdel-Ghaffar, Faten Aly Shoukry, Sascha Wilk Michelsen, Bolette Soborg, Thomas Blauenfeldt, Stellah Mpagama, Søren T. Hoff, Else Marie Agger, Ida Rosenkrands, Claus Aagard, Gibson Kibiki, Nabila El-Sheikh, Peter Andersen

**Affiliations:** 1 Statens Serum Institut, Denmark; 2 Department of Pathology, Hvidovre Hospital, Denmark; 3 Kilimanjaro Clinical Research Institute, Tanzania; 4 Molecular Immunology Unit, Al-Azhar University, Cairo, Egypt; 5 Animal Health Research Institute, Cairo, Egypt; 6 Biochemistry Department, Faculty of Science, Ain Shams University, Cairo, Egypt; 7 Abbasia Chest hospital, Ministry of Health, Cairo, Egypt; 8 Kibong’oto Infectious Diseases Hospital, Tanzania

## Abstract

There is a need for an improved vaccine for tuberculosis. ESAT-6 is a cardinal vaccine antigen with unique properties and is included in several vaccine candidates in development. ESAT-6 is also the core antigen in the IFN-γ release assays (IGRA) used to diagnose latent infection, rendering IGRA tests unspecific after vaccination. This challenge has prompted the development of a companion diagnostic for ESAT-6 based vaccines, an ESAT-6 free IGRA. We screened a panel of seven potential new diagnostic antigens not recognized in BCG vaccinated individuals. Three highly recognized antigens EspC, EspF and Rv2348c were identified and combined with CFP10 in an ESAT-6 free antigen cocktail. The cocktail was prepared in a field-friendly format, lyophilized with heparin in ready-to-use vacutainer tubes. The diagnostic performance of the ESAT-6 free IGRA was determined in a cross-validation study. Compared IGRA, the ESAT-6 free IGRA induced a comparable magnitude of IFN-γ release, and the diagnostic performance was on par with Quantiferon (sensitivity 84% vs 79%; specificity 99% vs 97%). The comparable performance of the ESAT-6 free IGRA to IGRA suggests potential as companion diagnostic for ESAT-6 containing vaccines and as adjunct test for latent infection.

Over the last two centuries tuberculosis (TB) is estimated to have killed one billion people, and remains among the world’s most lethal infectious diseases^1^. Neonatal BCG vaccination programs have reduced the incidence of severe childhood TB, but BCG has limited impact on adolescent and adult pulmonary TB, the driving force of the global TB pandemic^2^. It is evident that the current strategy to control TB is failing and the WHO post-2015 goal of a 90% reduction in the incidence of TB by 2035 will not be met without the introduction of new and efficacious vaccines for TB.

It is now recognized that the 10 kD small secretory antigenic target (ESAT-6) is a cardinal vaccine antigen essential for early bacterial containment and potentially also useful administered post exposure as a therapeutic vaccine^3^,^4^. ESAT-6 based vaccines have been extensively explored using both viral vector delivery and in adjuvanted subunit vaccines^5^,^6^,^7^. A major roadblock in the clinical development of ESAT-6 based vaccine candidates, is that ESAT-6 is contained in both the vaccine and as antigen in the IFN-γ release assay (IGRA) used to diagnose *Mycobacterium tuberculosis* (Mtb) infection^8^. For example, immunization with H56:IC31 leads to IGRA conversion in one in three vaccinees^9^, rendering IGRA tests unspecific. This problem has prompted the development of a companion diagnostic for ESAT-6 based vaccines, the ESAT-6 free IGRA. ESAT-6 based vaccines for TB are still far from implementation in vaccine programs; however, a tool to monitor infection in vaccinated individuals is urgently needed for trials exploring infection as a surrogate endpoint for TB disease. IGRA conversion occurs 10 times more frequently than disease, and prevention of infection (POI) trials can therefore be completed with less than a tenth of the sample size^10^,^11^. The fact that BCG vaccination reduces the risk of IGRA conversion in children further supports that POI can be utilized as a marker of biological effect^11^,^12^.

ESAT-6 (Rv3875) and its chaperone protein CFP10 (Rv3874) are the core antigens in the IGRA. These low molecular weight proteins are important virulence factors for Mtb, and the most immunodominant antigens thus far identified^13^. Their diagnostic specificity is attributed to their localization in the Region of Difference 1 (RD1) of the Mtb genome, which was deleted as part of the attenuation of BCG^14^. Neither ESAT-6 nor CFP10 alone are sufficient for reliable IGRA diagnosis of Mtb infection^15^. Omitting ESAT-6 from the IGRA leads to compromised diagnostic sensitivity, driven by both lower magnitude of IFN-γ release and failure of some infected individuals to recognize the remaining proteins. RD1 is part of the ESAT-6 system 1 (ESX-1) type VII secretion system which is associated with virulence and secretion of ESAT-6, CFP10, and other ESX-1 substrates^16^,^17^. One of the most distinctive aspects of ESX-1 secretion, is that the substrates are mutually dependent for secretion, suggesting a potential repertoire of candidate diagnostic antigens applicable for the ESAT-6 free IGRA beyond the 9 proteins encoded in RD1^18^. We focused on seven target antigens and antigen fragments based on established recognition patterns in infected humans and cattle (Table 1). One of these Ags, ESX-1 substrate protein C, (EspC, Rv3615c), has previously been identified as a highly recognized and Mtb specific antigen^13^,^19^,^20^,^21^,^22^. Other potential candidates include the ESX-1 associated EspJ (Rv3878)^23^,^24^, EspF (Rv3865)^25^,^26^,^27^,^28^,^29^, the RD1 encoded PPE68 (Rv3873)^23^, PE35 (Rv3872)^30^, EccD1 (Rv3877)^31^ and the RD7 encoded Rv2348^32^ (Table 1). By screening fragments in pools of three to seven peptides, we aimed to cover several antigens in the cocktail while retaining a low total number of peptides.

It is now evident that IFN-γ is only one of several potential biomarkers for detection of antigen specific responses for diagnostic purposes^33^. IP-10 is the leading alternative candidate to IFN-γ, and is expressed at 100-fold higher levels relative to IFN-γ, driving improved sensitivity over IGRA especially in immunosuppressed patients with HIV infection and in children^33^,^34^,^35^.

A challenge in assessing potential diagnostic antigens is the existence of conserved motifs present outside of deleted regions. This phenomenon has been described for regions of PPE68 and EspJ and can involve sequences not detected by BLAST homology searches using full-length protein sequences^24^,^36^. Such motifs can cause cross reactivity, emphasizing that the specificity of a potential diagnostic candidate requires experimental verification. We therefore screened selected fragments or full length of potential immunodiagnostic antigens sharing characteristics as immune recognition and specificity. A novel ESAT-6 free IGRA antigen cocktail was designed for complementarity to CFP10, and the diagnostic performance was cross validated in two populations head-to-head with ESAT-6 containing IGRA.

## Material and Methods

### Study participants

For initial antigen screening experiments, TB patients were included from Abbasia Chest hospital in Egypt and from Tasiilaq Hospital in East Greenland; healthy controls were included at Al-Azhar University medical schools teaching hospitals, Cairo, among 56 health care workers with negative Quantiferon Gold In-Tube, (Qiagen, Germany, QFT) test. Additionally, 18 Mtb infected controls (defined as recently exposed individuals with a positive QFT and no clinical suspicion of TB), were included from Tasiilaq; randomly selected among QFT positives identified through a large contact tracing study in East Greenland conducted by the National Health Authorities in 2011.

To determine IFN-γ release and cut off for the ESAT-6 free IGRA, we included patients with confirmed pulmonary TB from Abbasia Chest hospital, Cairo, Egypt and controls from Copenhagen, Denmark, who reported no travel or exposure history in a questionnaire through an advert (www.forsØgsperson.dk). The diagnostic algorithm was validated in 68 TB patients included at Kibong’oto Infectious Diseases Hospital, Tanzania and 35 endemic controls included among students and research staff at Kilimanjaro Christian Medical Centre and Kilimanjaro Clinical Research Institute, Moshi, Tanzania.

All participants provided written informed consent and the study was approved by relevant ethical committees. In Egypt by the Ethical Research Committee of the Ministry of Health and Population, Central Directorate for Research Health Development and at the Ethical Research Committee of Al-Azhar University, in Greenland by the Commission for Scientific Research in Greenland (approval No. 2012-4), in Tanzania by the Kilimanjaro Christian Medical College Research Ethics and Review Committee (CRERC IRB #408) as well as National Institute for Medical Research Ethical Committee (NIMR NatHREC #NIMR/HQ/R.8a/Vol.IX/1353), and in Denmark by the Capital Region of Copenhagen Committee on Health Research Ethics (approval No. H-3-2012-008) and by the Danish Data Protection Agency. All methods were performed in accordance with the relevant guidelines and regulations.

### Whole blood stimulation and IFN-γ and IP-10 measurements

For the antigen screening experiment, fresh whole blood was stimulated for 24 hours with pools of overlapping peptides from the selected antigen and antigen fragments (see Table 1) in a round bottomed 96 well tissue culture plate (NUNC, Denmark). Plasma was isolated and frozen for later analysis.

Synthetic overlapping peptides (20aa length) were produced by Genscript (NJ, USA). ESAT-6 and CFP10 peptides were designed to be identical as the peptides used in the QFT (15–25aa length)^15^. To facilitate simple sample collection and processing in the cross validation study, we developed a vacutainer based blood collection system. In brief, clean 4 ml vacutainer tubes (Greiner, Austria), were prepared by adding 25 ul mixture of 10 ug dissolved overlapping peptides and 10 IU heparin (antigen tube); 10 ug PHA (Sigma) and 10 IU heparin (positive control tube), or heparin alone (nil control tube). Tubes were snap frozen and lyophilized, hereafter tubes were recapped. Tubes were shipped and stored at +5C until the day of use where air was evacuated from the tube using a syringe and needle drawing 1.8 ml air (~vacuum for 1 ml blood draw). IFN-γ and IP-10 levels were determined using ELISA as described previously^37^ except for the cross-validation study wherein IFN-γ levels were determined using the QFT ELISA (Qiagen, De). In the initial screening a responder was defined as antigen-specific release >50 pg/ml IFN-γ as suggested in the litterature^38^.

### Statistics

Biomarker expression levels were compared using non-parametric methods (Kruskal-Wallis test). Diagnostic potential was assessed using Receiver operating curve (ROC) analysis, which also was used to guide the determination of cut offs set at the level rendering highest sensitivity at >95% specificity. Difference in test positivity was assessed using McNemars test, after exclusion of indeterminate responders. Agreement was assessed using Cohens Kappa. Results were generated using SAS 9.4 (SAS institute, NC, USA), graphs were prepared using GraphPad Prism 6 (GraphPad Software Inc, USA).

## Results

### Antigen screening and design of ESAT-6 free IGRA

Ten RD1, RD7 and ESX1 related antigens were screened using pools of overlapping peptide spanning either the whole protein or selected regions previously shown to contain epitope hot spots recognized in humans (Table 1).

**Figure 1:**
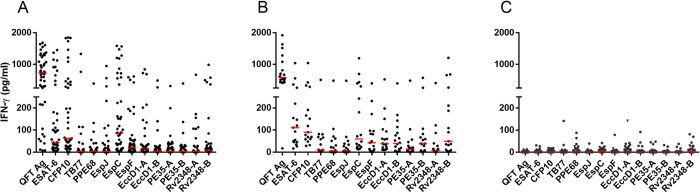
IFN-γ release to QFT antigens, 10 screened Mtb specific antigens and selected regions within antigens. Diluted whole blood from 48 patients with TB (**A**) 34 Egyptian and 14 from Greenland), 18 Mtb infected controls from Greenland (**B**) and 56 uninfected controls from Egypt (**C**) was stimulated 24 hours with overlapping peptides (as described in Table 1) in 200 ul volume. IFN-γ release was determined using in-house ELISA and presented following subtraction of IFN-γ release in an unstimulated control well. Median values are indicated in red.

IFN-γ release from antigen stimulated whole blood culture in 48 patients with microbiologically or microscopy confirmed pulmonary TB (34 from Egypt and 14 from Greenland), 18 Mtb infected controls (Greenland), and 56 controls with negative IGRA (Egypt) were compared in terms of magnitude of IFN-γ release, and responder frequency (Fig. 1 and Table 2). ESAT-6, CFP10 and EspC were the most frequently recognized (>40% responders of TB and LTBI), Rv2348-B and EspF were less frequently recognized (15–40%) whereas TB7.7, EspJ, EccD1, PE35 and the N-terminal of Rv2348c (Rv2348-A) were infrequently detected. The group of LTBI donors had a wider antigen repertoire compared to the TB patients, whom almost exclusively focused on ESAT-6, CFP10 and EspC. There were no significant differences in the antigen recognition repertoire among TB patients from Greenland and Egypt (data not shown).

We explored the complementarity of the antigens by combination in the pooled group of Mtb infected and TB patients (n = 66) and controls (n = 56) (Table 3). Among infected and TB patients, ESAT-6 recognizing donors all co-recognized CFP10 except in three (5%) individuals. In contrast, EspC had a different recognition profile, detecting 11% (7/66) not recognized by CFP10 and 12% (8/66) not picked up by ESAT-6. EspF and Rv2348-B were most frequently co-recognized with CFP10 and EspC, adding to the overall IFN-γ release in the population and complimenting the other antigens in those participants only recognizing one of two major antigens. There was no specific pattern associated with active or latent TB (data not shown). EccD1-A and the less frequently detected antigens did not benefit the coverage in the population (Table 3 and data not shown). In the controls, none of the explored antigens were recognized in more than 2 donors, confirming their specificity. Therefore, we defined the ESAT-6 free IGRA antigen cocktail comprising overlapping peptides from CFP10, EspC, EspF and Rv2348-B.

### *In silico* assessment of antigen and antigen fragment specificity

The individually assessed peptides as well as the full protein sequence of EspC, EspF and Rv2348-B were compared to ESAT-6 and CFP10 for cross reactivity with *Mtb* complex and environmental and other non-tuberculous mycobacteria (NTM) using Basic Local Alignment Search Tool (BLAST, http://blast.ncbi.nlm.nih.gov/Blast.cgi, supplement 1). All assessed antigen regions shared full sequence homology with the three members of *MTb* complex. Overall EspC, Rv2348c and EspF showed less sequence homology with environmental- or other mycobacteria as compared to ESAT-6 and CFP10. Of note, the two NTM strains known to cause most false positive IGRA results (*M. kansasii* and *M. marinum)* also shared the highest sequence homology among the environmental strains, however levels were lower (66–83%) as compared to ESAT-6 and CFP10 (>95%). *M. szulgai*, the third clinically relevant NTM known to cause positive IGRA results, did not share sequence homology for any of the selected antigen regions in the ESAT-6 free IGRA.

### Development of a diagnostic algorithm for the ESAT-6 free IGRA

To facilitate easy sample collection and processing in the field, the ESAT-6 free peptide cocktail was prepared in heparinized vacutainer tubes. A parallel in-house QFT tube containing the QFT peptide cocktail^15^ was prepared to benchmark the in-house vacutainer tube system. The magnitude of responses, diagnostic specificity and cut offs for positive ESAT-6 free IGRA was determined in 74 patients with culture confirmed active TB from Cairo, Egypt and 100 unexposed controls in Copenhagen, Denmark (Table 4). Patients were older and more frequently male compared to controls and had more frequent co-morbidity: 18 (23%) reported diabetes, three bilharzias and one HCV infection. Sixty-three of 74 (85%) TB patients were QFT positive, 3/100 (3%) of controls has positive IGRA and no apparent exposure or travel history.

**Figure 2:**
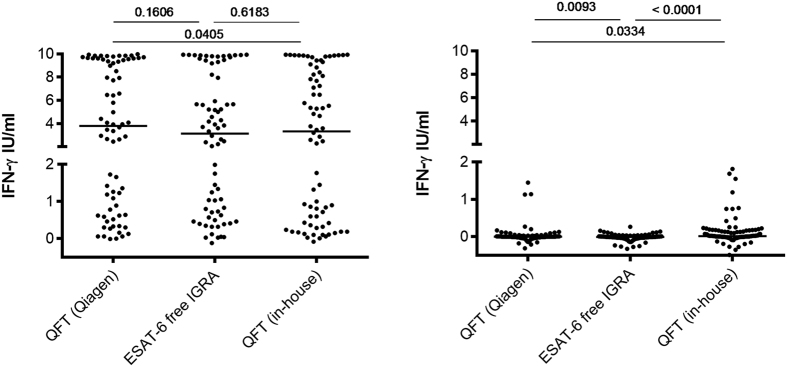
Antigen specific release of IFN-γ in patients (left, n = 74) and controls (right, n = 100) stimulated with QFT and ESAT-6 free IGRA cocktails. One ml whole blood was stimulated 18–24 h in antigen coated heparinized vacutainer tubes (in-house and QFT (Qiagen)), antigen specific IFN-γ release was determined using QFT ELISA (Qiagen) and is presented subtracting unspecific release (nil) in heparinized vacutainer tubes with no peptides (in-house and QFT (Qiagen)). Kruskal-Wallis test.

The magnitude of IFN-γ release in the ESAT-6 free IGRA antigen tubes was comparable to the QFT (Qiagen) in samples from TB cases (median 3.1 IU/ml (IQR 0.6–8.5 IU/ml) vs 3.8 (0.6–9.5 IU/ml), p = 0.161); but significantly lower in the QFT (in-house) tubes (median 3.3 IU/ml (IQR 0.4–8.5 IU/ml, p = 0.041) compared to QFT (Qiagen) (Fig. 2). In the controls, responses were significantly lower in the ESAT-6 free IGRA compared to QFT (Qiagen) and QFT (in-house) although this difference was small and clinically insignificant (median difference 0.01 UI/ml). Interestingly, 3% (3/100) and 7% (7/100) of the controls responded >0.5 IU/ml in the QFT (Qiagen) and QFT (in-house) tubes, respectively.

**Figure 3:**
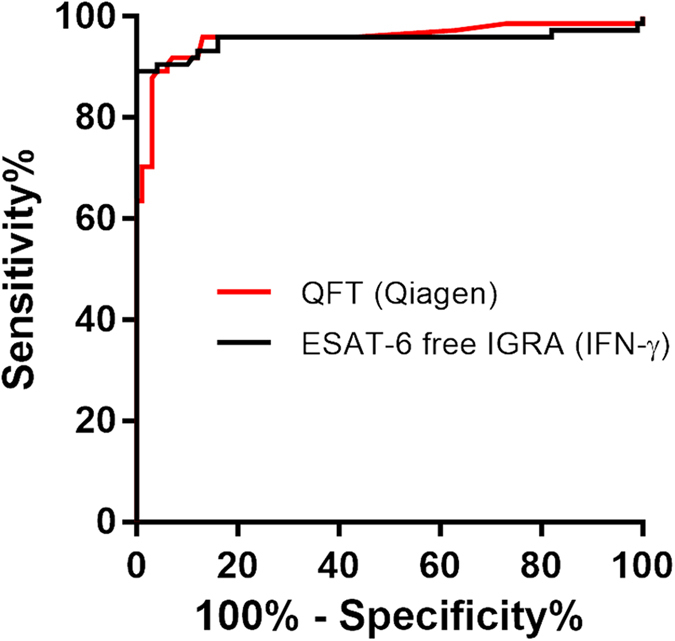
ROC Curve analysis comparing the diagnostic potential of ESAT-6 free IGRA and QFT (Qiagen). Analysis included samples from 74 patients and 100 controls. The Area Under the Curve reflects the antigen cocktails’ ability to differentiate between cases and controls. AUC for QFT (Qiagen) 0.96 (95% CI 0.92–0.99), ESAT-6 free IGRA (IFN-γ) 0.95 (95% CI 0.91–0.99). There was no significant difference between the ROC curves.

The diagnostic potential of the ESAT-6 free IGRA was assessed in ROC curve analysis (Fig. 3). Here, the ESAT-6 free IGRA had a very high area under the curve (AUC) 0.95 (95% CI 0.91–0.99) not significantly different from QFT (Qiagen) 0.96 (95% CI 9.92–0.99).

ROC curve analysis suggested a cut off between 0.15 IU/ml and 0.30 IU/ml IFN-γ for positive ESAT-6 free IGRA (sensitivity 89%, specificity 99–100%) and confirmed the ≥0.35 IU/ml as optimal for the QFT (Qiagen) (sensitivity 84%, specificity 97%) (Fig. 3). To ensure optimal comparability with the QFT (Qiagen) test in the cross validation, we defined a diagnostic algorithm for ESAT-6 free IGRA: a test was defined as positive if antigen response subtracted the nil response was ≥0.25 IU/ml for IFN-γ irrespective of PHA mitogen control; negative if antigen response subtracted with nil response was <0.25 IU/ml and PHA mitogen control ≥0.5 IU/ml for IFN-γ; indeterminate if negative and PHA mitogen control was below cut off.

IP-10 responses in the ESAT-6 free IGRA were assessed in parallel to IFN-γ (see supplement 2). Antigen-specific IP-10 release was 36 fold higher as compared to IFN-γ (p < 0.0001, Kruskal-Wallis) and the ROC curve analysis rendered AUC for the IP-10 based ESAT-6 free IGRA at 0.98 (95% CI 0.95–1.00)), which was comparable to the AUC for QFT (Qiagen) with IFN-γ (AUC = 0.96 (95% CI 0.92–0.99))

### Validation cohort

The diagnostic accuracy of the ESAT-6 free IGRA was compared to QFT (Qiagen) in two independent cohorts of 68 TB patients and 35 endemic controls from Tanzania. TB patients all had positive x-ray signs suggestive of pulmonary TB and 94% (59/62, (6 not done)) were confirmed with sputum smear. All had received treatment for less than 4 weeks, of whom 62% (42/68) for less than two weeks. TB patients were older, median age 38 years (range 16–68) and predominately male 78% (53/68), compared to endemic controls who were younger, median age 33 years (24–47) and predominately female 63% (22/35). Eleven TB patients were tested positive for HIV infection, 4 had a CD4 T cell count available (66, 175, 250 and 1031 cells/μl). In the validation cohort, ESAT-6 free IGRA identified 84% as positive with IFN-γ and 74% with IP-10, compared to 79% for QFT (Qiagen) with both IFN-γ and IP-10 (p > 0.563). One patient had concordant indeterminate test result. Among the endemic controls the IFN-γ based tests both detected 49% (17/35) as positive whereas IP-10 detected 43% with the QFT cocktail (ns.) and 37% with the ESAT-6 free IGRA (p = 0.046, Table 5).

Agreement was high between ESAT-6 free IGRA and QFT 82% (55/67, *k* = 0.392) in the TB group and 73% (27/35, *k* = 0.543) in the endemic control group; 12 (18%) were discordant in the TB group and 8 (23%) in the endemic control group (supplement 3). Combining ESAT-6 free IGRA with standard IGRA defining test positivity as either/or test being positive, significantly improved sensitivity to 91% (p < 0.005). The majority of participants with discordant results had IFN-γ release levels for both tests in the “grey zone” around the cut off (0.2–0.70 IU/ml) where highest analytical variability is to be expected. We observed no impact of HIV infection on the positivity rate of either test among the TB patients (QFT (IFN-γ) 81% (9/11) and ESAT-6 free IGRA 72% (8/11), negative results were not found among the three HIV infected with an available CD4 T cell below 500 cells/μl.

## Discussion

In this study, we developed an immunodiagnostic test intended for latent Mtb infection as a companion diagnostic for ESAT-6 containing vaccines. We screened a panel of presumed specific but little explored antigens for immunodiagnostic potential in patients with active TB and latently infected persons. The three most promising antigens were selected based on magnitude of IFN-γ release and complementarity to CFP10, and were combined in a new ESAT-6 free antigen cocktail comprising CFP10 and fragments of EspC, EspF and Rv2348c (Table 1). The antigen cocktail was prepared in a vacutainer tube based system allowing standardized and field friendly assessment. The ESAT-6 free IGRA generated IFN-γ and IP-10 release in comparable magnitude and demonstrated diagnostic performance on par with the QFT test.

We selected our seven new target antigens and antigen fragments based on *in silico* predicted specificity and/or established recognition patterns in infected humans and cattle (Table 1). In contrast to the standard approach in antigen screening studies using full length of the antigen^39^,^40^, we focused our investigations on smaller fractions of antigens, hereby allowing the incorporation of peptides from several antigens in the cocktail while retaining a low total number of peptides. Our findings confirm ESAT-6, CFP10 and the N-terminal of EspC as highly recognized^19^,^20^,^21^, and suggest EspF and Rv2348c as novel potential adjunct antigens for diagnostic and vaccine purposes.

EspC has previously been identified as a highly recognized and Mtb specific antigen^13^,^19^,^20^,^21^,^22^. The majority of T cell epitopes are concentrated in the N-terminal half, which was also assessed herein (ref. 20, Ruhwald unpublished). Despite being based on only 4 peptides, the EspC fragment was recognized at the same level as the 7 peptides spanning ESAT-6. Compared to ESAT-6 and CFP10, the BLAST screening of EspC shoved less strain homology to environmental mycobacteria further supporting the diagnostic potential of the antigen. To further improve the response level and coverage in the population, we complemented CFP10 and EspC with the second and third ranking novel candidates EspF and Rv2348c. EspF was assessed in a small 3 peptide pool covering the C-terminal part of the molecule. EspF is a small antigen encoded upstream of RD1 and a paralogue of EspC, sharing 52% similarity and 37% identity and considered an important virulence factor for Mtb^25^,^26^,^27^,^28^. Vis-à-vis EspC, EspF requires a functional ESX1 system for secretion, ensuring diagnostic specificity in BCG vaccinated. The immunodiagnostic potential of EspF was first demonstrated in cattle using the *M*.*bovis* equivalent Mb3895^28^, and human recognition was very recently shown as part of a larger screening study^29^. The third new antigen Rv2348c is a hypothetical protein with unknown function. Rv2348c, is located in the RD7 region, shown to be absent in *M*.*bovis* and BCG^14^. The gene is highly transcribed *in-vitro*^32^ and the protein has been identified in proteome studies^41^.

We demonstrated that Rv2348c and EspF are recognized in diverse populations comprising participants from Inuit, North African and sub-Saharan populations, suggesting that the antigens and antigen fragments include epitopes which are recognized in the context of a wide range of HLA-II haplotypes, similar to the multiple promiscuous epitopes previously described for ESAT-6, CFP10 and EspC^13^,^20^,^42^. PPE68, EccD1, EspJ and PE35 were less frequently recognized in this study compared to previous reports^21^,^30^,^39^, suggesting that our selected regions did not contain the immunogenic epitopes and warrants further exploration.

IGRA tests are primarily tools developed to guide preventive treatment of infected individuals at risk of developing TB^43^. Due to the lack of a gold standard for infection, active TB is often used as a model to evaluate test performance^15^,^44^. The diagnostic performance of IGRAs has been extensively assessed and meta-analysis data have established the diagnostic sensitivity in TB patients without comorbidity to 80% and very low false-positive rate in healthy individuals from low endemic regions (97%)^8^. Our results confirm these numbers and suggest that the ESAT-6 free IGRA has comparable diagnostic performance, allowing us to advance the validation of the assay in larger studies, in preparation for prevention of infection trial of H56:IC31 and other ESAT-6 based vaccines. It is a limitation in our design that the Tanzanian TB patients were not confirmed by culture or molecular methods. NTM co-infection could therefore bias the findings in the validation cohort, however the absence of sequence homology with NTM in the selected antigens in the BLAST analysis and the experimental confirmation in culture positive TB vases in the Egypt cohort suggest this bias to be limited.

The agreement between ESAT-6 free IGRA using IFN-γ and QFT was high (~80%) in all cohorts. IP-10 was highly expressed in terms of magnitude of release and showed comparable diagnostic potential in the ROC curve analysis, wherefore it was unexpected that IP-10 deemed fewer endemic controls positive in the validation cohort with the ESAT-6 free IGRA cocktail. The high agreement between IP-10 and IFN-γ based IGRA tests is in line with previous reports (reviewed in ref. 33) and calls for further studies to confirm if these findings. Some discordance is expected in a complex biological assay system as IGRA, and discordance is a well known phenomenon with serial or parallel IGRA testing^45^. Discordance between biomarkers as well as antigen cocktails can be utilized to increase the number of positive responders by classifying a positive outcome if at least one test is positive. This was also the case for this study, where we observed a significantly improved sensitivity from 79% to 91% (61/67) by combining IGRA and ESAT-6 free IGRA (p < 0.005).

Major limitations to the IGRAs in their current form is a poor positive predictive value for development of disease and insufficient detection of infection in immunosuppressed individuals (especially HIV infected and in young children)^8^,^46^,^47^. By selecting antigens based on ESAT-6 like characteristics (e.g. virulence and expression during exponential growth), it is unlikely that the ESAT-6 free IGRA will improve the predictive value. However, the findings suggest that ESAT-6 can be added to the ESAT-6 free IGRA cocktail to improve assay performance in the high risk groups as an alternative approach to the combination approach described above.

To standardize format and improve field friendliness, we prepared lyophilized antigen cocktails in vacutainer tubes. The tube system performed remarkably well in the ESAT-6 free IGRA, generating IFN-γ release on par with responses in the QFT tubes. However, when preparing the QFT antigen cocktail in in-house tubes, this was less specific (using both IFN-γ and IP-10), suggesting an unspecific activation in the whole blood sample. This activation is likely not caused by the tube format, but seemingly associated with the preparation of ESAT-6 or TB7.7p4 in the tube. Whether this phenomenon stems from too much peptide added, peptide clumping or peptide-heparin interactions is a question for further study, but suggests that the performance of the in-house tubes could be further improved by elimination of this factor.

In conclusion, we have established a novel immunodiagnostic test for latent Mtb infection intended as a companion diagnostic for ESAT-6 containing vaccines. The ESAT-6 free IGRA generated IFN-γ and IP-10 release in comparable magnitude to the QFT test, and demonstrated diagnostic performance on par with the QFT test.

## Additional Information

**How to cite this article**: Ruhwald, M. *et al*. Introducing the ESAT-6 free IGRA, a companion diagnostic for TB vaccines based on ESAT-6. *Sci. Rep.* **7**, 45969; doi: 10.1038/srep45969 (2017).

**Publisher's note:** Springer Nature remains neutral with regard to jurisdictional claims in published maps and institutional affiliations.

## Supplementary Material

Supplementary Information

## Figures and Tables

**Table 1:** Overview of screened antigens and peptides.

Name	Rv No	Function	Protein length	Fraction assessed	No. of peptides	Refs
ESAT-6	Rv3875	RD1, ESX1 substrate	95	1–95	7	13,48
CFP10	Rv3874	RD1, ESX1 substrate	100	1–100	6	10,49
TB7.7	Rv2654c	RD11, unknown	81	1–81	6	13,36
PPE68	Rv3873	RD1, PPE family	368	147–204	5	23
EspJ	Rv3878	RD1, ESX1 associated protein	280	122–189	5	23,24
EspC	Rv3615c	ESX1 substrate	103	54–103	4	13,19, 20, 21, 22
EspF	Rv3865	ESX1 associated protein	103	9–44	3	25, 26, 27, 28
EccD1-A	Rv3877	RD1, ESX1 protein (predicted surface regions)	511	218–282	5	31
EccD1-B				442–511	5	
PE35-A	Rv3872	RD1, PE family	99	1–59	4	30
PE35-B				53–99	3	
Rv2348-A	Rv2348c	RD7, unknown	108	1–64	5	14
Rv2348-B				55–108	5	

**Table 2:** Responder rates in TB patients, LTBI and controls. A responder is defined as antigen-specific release >50 pg/ml IFN-γ.

Antigen, n (%)	TB (n = 48)	LTBI (n = 18)	Ctrl (n = 56)
ESAT-6	19 (40)	9 (50)	0 (0)
CFP1	27 (56)	11 (61)	0 (0)
TB7.7	3 (6)	1 (6)	1 (2)
PPE68	1 (2)	0 (0)	3 (5)
EspJ	5 (10)	0 (0)	1 (2)
EspC	22 (46)	8 (44)	1 (2)
EspF	11 (23)	6 (33)	2 (4)
EccD1-A	6 (13)	4 (22)	2 (4)
EccD1-B	2 (4)	4 (22)	1 (2)
PE35-A	2 (4)	1 (6)	1 (2)
PE35-B	3 (6)	3 (17)	1 (2)
Rv2348-A	5 (10)	0 (0)	0 (0)
Rv2348-B	6 (13)	8 (44)	2 (4)

**Table 3:** Overview of antigen complementarity and recognition patterns in TB and LTBI groups and controls.

	ESAT-6	CFP10	EspC	EspF	Rv2348-B	EccD1-A	n (%)
TB and LTBI (n = 66)	−	−	−	−	−	−	19 (29)
	−	−	−	−	+	−	2 (3)
	−	−	+	−	−	−	2 (3)
	−	−	+	−	+	−	1 (2)
	−	−	+	+	−	−	1 (2)
	−	+	−	−	−	−	8 (12)
	−	+	−	−	+	−	1 (2)
	−	+	+	−	−	−	2 (3)
	−	+	+	+	−	−	1 (2)
	−	+	+	+	+	−	1 (2)
	+	−	+	−	−	−	2 (3)
	+	−	+	+	−	−	1 (2)
	+	+	−	−	−	−	5 (8)
	+	+	−	+	+	−	1 (2)
	+	+	+	−	−	−	6 (9)
	+	+	+	−	+	+	1 (2)
	+	+	+	+	−	−	4 (6)
	+	+	+	+	−	+	1 (1)
	+	+	+	+	+	−	3 (5)
	+	+	+	+	+	+	4 (6)
n (%)	28 (42)	38 (58)	30 (45)	17 (26)	14 (21)	6 (9)	
Control (n = 56)	−	−	−	−	−	−	52 (93)
	−	−	−	−	+	−	2 (4)
	−	−	−	+	−	+	1 (2)
	−	−	+	+	−	−	1 (2)
n (%)	0 (0)	0 (0)	1 (2)	2 (4)	2 (4)	1 (2)	

Response patterns to ESAT-6, CFP10, EspC, EspF, Rv2348B and EccD1-A antigens pools were calculated for each individual with a responder defined as antigen-specific release >50 pg/ml IFN-γ (+). Recognition pattern is described with +/− and the number of donors with the specific antigen recognition pattern is summarized in the right column as number of volunteers with the specific pattern and % of total.

**Table 4:** Baseline table, cases and controls used to determine cut off in cross validation study.

	TB cases	Controls
**n**	74	100
**Male sex, n (%)**	66 (86)	34 (34)
**Age, median (range)**	35 (28–52)	29 (24–42)
**Country of birth**		
Egypt	74 (100)	0 (0)
Denmark	0 (0)	89 (89)
Other low endemic	0 (0)	11 (11)
**BCG vaccinated**		
yes	37 (50)	58 (58)
no	0 (0)	23 (23)
unknown	37 (50)	19 (19)
**Co-morbidity**		
None	52 (70)	97 (97)
Yes	22 (30)	2 (2)
unknown	0 (0)	1 (1)
**Weeks treatment**		
0 weeks	52 (70)	—
<2 weeks	22 (30)	—
**Exposure**		
**Smear**		
positive	71 (96)	—
negative	3 (4)	—
n.d.	0 (0)	—
**x-Ray**		
positive	64 (87)	—
negative	7 (9)	—
n.d.	3 (4)	—

**Table 5:** Test results in validation cohort, patients with confirmed active TB (n = 68) and endemic controls (n = 35) from Tanzania.

		QFT (IFN-γ)	QFT (IP-10)	ESAT-6 free IGRA (IFN-γ)	ESAT-6 free IGRA (IP-10)
Patients (n = 68)	Positive	54 (79)	54 (79)	56 (84)	50 (74)
	Negative	12 (20)	12 (20)	11 (16)	17 (25)
	Indeterminate	1 (1)	1 (1)	1 (1)	1 (1)
Endemic controls (n = 35)	Positive	17 (49)	15 (43)	17 (49)	13 (37)*
	Negative	18 (51)	20 (57)	18 (51)	22 (63)
	Indeterminate	0 (0)	0 (0)	0 (0)	0 (0)

IFN-γ and IP-10 levels were determined using ELISA and deemed positive, negative and indeterminate using cut offs determined in a training set 0.25 IU/ml for ESAT-6 free IGRA (IFN-γ), 1.0 ng/ml for ESAT-6 free IGRA (IP-10) and 1.3 for QFT (IP-10). *p = 0.046, McNemars test compared to QFT (IFN-γ), all other comparisons to QFT (IFN-γ) were non-significant.
