# Supplementary material for: Introducing the ESAT-6 free IGRA, a companion diagnostic for TB vaccines based on ESAT-6
Source: Sci Rep. 2017 Apr 7;7:45969. doi: 10.1038/srep45969 (PMC5384086; doi:10.1038/srep45969)
Supplement: Supplementary Information [file srep45969-s1.doc]

**Online supplement 1-3.**

Title

Introducing the ESAT-6 free IGRA, a companion diagnostic for TB vaccines based on ESAT-6

Authors

Morten Ruhwald^1*^, Lena de Thurah^1,2^, Davis Kuchaka^3^, Mustafa Raafat^4,8^, Ahmed M. Salman^7^, Abdel-Rahman Abdel-Ghaffar^7^, Faten Aly Shoukry^5^, Sascha Wilk Michelsen^1^, Bolette Soborg^1^, Thomas Blauenfeldt^1^, Stellah Mpagama^6^, Søren T. Hoff^1^, Else Marie Agger^1^, Ida Rosenkrands^1^, Claus Aagard^1^, Gibson Kibiki^3^, Nabila El-Sheikh^4^, Peter Andersen^1^

**Online supplement 1.**

Homology search for cross reactivity of EspC, EspF and Rv2348c with Mtb complex, environmental and other non-tuberculous mycobacteria using Basic Local Alignment Search Tool (BLAST, http://blast.ncbi.nlm.nih.gov/Blast.cgi). Numbers indicate % homology, red tones <50%, blue >50% sequence homology.

**Online supplement 2**

IP-10 responses and diagnostic potential of the ESAT-6 free IGRA using IP-10. IP-10 responses in the ESAT-6 free IGRA were assessed in parallel to IFN-γ. Antigen-specific release was 5.5ng/ml (2.3-11.8ng/ml) in cases, median 36 fold higher compared to IFN-γ (p<0.0001), and 0.0 ng/ml (-0.1-0.2ng/ml in controls (p>0.0001). ROC curve analysis rendered AUC for the IP-10 based ESAT-6 free IGRA comparable to QFT (Qiagen, IFN-γ) and suggested a cut off at 1.0ng/ml (Sensitivity 88%, Specificity 98%).

**Online supplement 3.**

QFT and ESAT-6 free IGRA (IFN-γ) test concordance in the validation cohort, patients (n=67, left), endemic controls (n=35, right). One patient with concordant indeterminate test result was excluded from the left table.

| IGRA | ESAT-6 free IGRA | | | |  | IGRA | ESAT-6 free IGRA | | | |
| --- | --- | --- | --- | --- | --- | --- | --- | --- | --- | --- |
|  |  | + | - |  |  |  |  | + | - |  |
|  | + | 49 | 7 | 56 |  |  | + | 13 | 4 | 17 |
|  | - | 5 | 6 | 11 |  |  | - | 4 | 14 | 18 |
|  |  | 54 | 13 | 67 |  |  |  | 17 | 18 | 35 |

Kappa 0.392 Kappa 0.542
